# Proposed methods for reviewing the outcomes of health research: the impact of funding by the UK's 'Arthritis Research Campaign'

**DOI:** 10.1186/1478-4505-2-4

**Published:** 2004-07-23

**Authors:** Stephen R Hanney, Jonathan Grant, Steven Wooding, Martin J Buxton

**Affiliations:** 1 Health Economics Research Group, Brunel University, Uxbridge, Middlesex UB8 3PH, UK; 2 RAND Europe, Grafton House, Cambridge CB5 8DD, UK

## Abstract

**Background:**

External and internal factors are increasingly encouraging research funding bodies to demonstrate the outcomes of their research. Traditional methods of assessing research are still important, but can be merged into broader multi-dimensional categorisations of research benefits. The onus has hitherto been on public sector funding bodies, but in the UK the role of medical charities in funding research is particularly important and the Arthritis Research Campaign, the leading medical charity in its field in the UK, commissioned a study to identify the outcomes from research that it funds. This article describes the methods to be used.

**Methods:**

A case study approach will enable narratives to be told, illuminating how research funded in the early 1990s was (or was not) translated into practice. Each study will be organised using a common structure, which, with careful selection of cases, should enable cross-case analysis to illustrate the strengths of different modes and categories of research. Three main interdependent methods will be used: documentary and literature review; semi-structured interviews; and bibliometric analysis. The evaluative framework for organising the studies was previously used for assessing the benefits from health services research. Here, it has been specifically amended for a medical charity that funds a wide range of research and is concerned to develop the careers of researchers. It was further refined in three pilot studies. The framework has two main elements. First, a multi-dimensional categorisation of benefits going from the knowledge produced in peer reviewed journal articles through to the health and potential economic gain. The second element is a logic model, which, with various stages, should provide a way of organising the studies. The stock of knowledge is important: much research, especially basic, will feed into it and influence further research rather than directly lead to health gains. The cross-case analysis will look for factors associated with outcomes.

**Conclusions:**

The pilots confirmed the applicability of the methods for a full study which should assist the Arthritis Research Campaign to demonstrate the outcomes from its funding, and provide it with evidence to inform its own policies.

## Background

### The growing concern for the benefits from health research to be studied

Health research funding bodies are under increasing pressure to demonstrate the outcomes, or benefits, of the research that they fund [1-6]. Traditional peer review of research focussed on the outputs in terms of journal articles, the training of future researchers and the development of careers. These are still seen as important, but in some analyses they have been merged into broader multi-dimensional categorisations of the benefits from health research [7,8].

The onus hitherto has been on public sector funding bodies. There is a general recognition in the UK, however, of the importance of the role of the medical charities: they fund approximately one third of UK medical research – a level 'unparalleled elsewhere in the world and nor is it found in other areas of science' [9]. Therefore, in an era of accountability, public involvement in research issues and growing competition for contributions, some medical charities see the virtue in being able to demonstrate the outcomes of the research they fund. Not all the pressures, however, are external and some funding bodies, including the Wellcome Trust, which is an endowment and not collection-based charity, are being pro-active in their attempts to identify and track the outcomes of the research they fund. One factor relevant for both public sector and charity funding bodies is the recognition that assessing the benefits from their research may assist in identifying research strategies most likely to produce benefits [2,7,10,11]. Concerns such as those above led a UK medical charity, the Arthritis Research Campaign (ARC), to approach RAND Europe with the idea of conducting an assessment of the long-term outcomes from research that they have funded.

The purpose of this paper is to set out the aims of the study and the methods being adopted. In particular, it will show how an existing generic approach to the assessment of benefits from health research [7,12] has been adapted to meet the needs of this specific study. After providing the background to the study, the paper describes the methods to be adopted. These were initially agreed after a consultative phase in which the evaluative framework was refined on the basis of interviews with six key actors who have played various roles within ARC and advice from ARC's Development Committee, which acts as the steering group for this project. They were then confirmed following a pilot stage in which three case studies were conducted; some examples from the pilot studies, which endorsed the feasibility of the proposed approach, will be given to illustrate the account of the methods.

### The increasing attention on musculoskeletal conditions and the role of the Arthritis Research Campaign (ARC)

Attention is being drawn to the increasing scale of the burden of musculoskeletal conditions, and the associated costs, in various ways including through the establishment of the Bone and Joint Decade 2000–2010 and the recent collaborative report with the World Health Organization (WHO) [13]. At the same time, there is a realisation that the benefits of research in this area are sometimes less immediately apparent than in some other fields. For example, two classic studies of the economic benefits from biomedical research [14,15] both highlighted arthritis as an area where research and higher medical care expenditure may have comparatively little impact on mortality. Furthermore, one recent attempt to put a monetary value on the benefits from health research in Australia [16] adapted a method developed in the USA [17] and again demonstrates the difficulties of undertaking such analysis in the musculoskeletal field. These observations might suggest that a more careful and wide-reaching assessment of benefits from research is particularly needed in the field of arthritis.

ARC is the leading medical charity in this field in the UK and one of the largest collection-based medical charities in the UK. The Research Outputs Database (ROD) records the funding acknowledgements on all UK biomedical papers contained on the citations indices of the Institute for Scientific Information [18]. Analysis conducted on ROD reveals ARC to be, 'in a dominant position within the UK in the arthritis subfield' [19]. ARC's funding is associated with more arthritis publications than that from either the Medical Research Council (MRC) or the Wellcome Trust. It spent almost £22 million in the year 2001–2002; its major aim 'is to support the highest quality research into the cause, cure and treatment of arthritis and musculoskeletal diseases' [20]. It adopts a variety of funding modes for a range of types of research including its support of two research centres. The preliminary interviews, described above, highlighted the importance of the work by Marc Feldmann and Sir Ravinder Maini at one of these, the Kennedy Institute of Rheumatology in London, in developing anti-Tumour Necrosis Factor (anti-TNF) therapy as an effective treatment for rheumatoid arthritis and other autoimmune diseases. The pair won the 2003 Albert Lasker Award for Clinical Medical Research for this discovery.

### Objectives of the evaluation/research questions

Within the general climate of an increased emphasis on the outcomes from research, four main objectives were specified for the particular study described in this article:

• Review and document outcomes for ARC research grants

• Illustrate the strengths and weaknesses of different modes of research funding

• Identify factors associated with translation of research, and attempt to develop 'early indicators' of likely successful translation

• Identify 'good news stories' and vignettes of the research process for use by ARC in public engagement and fund raising activities.

## Methods

### Rationale for using a case study approach

Traditional methods of peer review have long been favoured by medical research funding bodies for evaluating research, but bibliometric methods have had a variable history. An early move by the National Institutes of Health (NIH) to establish a publications' database was cancelled by the 1980s 'as too expensive for the management information it produced' [21]. The MRC in the UK reviewed the possibility of making greater use of bibliometric measures to inform per review of major long term programmes, but the steering group established to oversee the review concluded that, 'bibliometric analysis would not add sufficient value to peer review to be worthwhile routinely and should not be introduced into MRC procedures' [22]. Nevertheless, there are circumstances where bibliometric analysis can provide research funding bodies with useful information [19] and, as discussed below, they can be incorporated into broader case studies. On its own, however, it is unlikely to provide much information about the longer-term outcomes from research funding.

The Economic and Social Research Council (ESRC) in the UK commissioned a project to identify the impact of their research on non-academic audiences. It involved tracing the activity of the participating researchers after their projects ended and mapping the networks of researchers and relevant non-academic users and potential beneficiaries [23]. The study concluded that the preferable way to determine and assess the existence of impacts of socio-economic research on non-academic audiences 'is through detailed, project-by-project qualitative analysis' [23]. Such an approach probably entails adopting a case study approach, and there is a long history of applying the case study approach to examine the utilisation of research [24]. Indeed, where the emphasis is on demonstrating the outcomes from health research, a case study approach has mainly been used [7,25] and been recommended for use in future studies [4,26,27].

Case studies will enable narratives or stories to be told to illuminate how the research funded in the early 1990s was translated (or not) into practice; each case, therefore, could potentially provide an illustrative example of the outcomes from ARC research. Furthermore, the planned 16 case studies will be based on a variety of modes of ARC research funding and types of research. They will also be organised using a common structure. This should enable cross-case study analysis to demonstrate (via illustrative case studies) the strengths and weaknesses of different modes of funding and categories of research. It should also facilitate the identification of factors associated with the translation of research, perhaps through various phases, into policies, products, and clinical practice that produce a health gain.

The evaluation framework described below was developed in a way that incorporates previous experience and knowledge on these issues [7,12,24,26,28]. This should ensure that questions are asked about a range of factors that previous experience suggests are likely to be related to the translation of research. Additionally, because the evaluation framework includes a multi-dimensional categorisation of benefits from research, the full range of outputs and outcomes relevant to different types of research, and modes of funding, will be looked for in the studies. The case studies will, in part, be conducted to see if they produce evidence consistent with existing hypotheses about factors linked to the translation of research and the role of different modes of research funding and types of research. But they will also be exploratory and should allow the generation of new hypotheses, particularly ones specifically relevant for research funding from a medical charity.

### Timescale

In deciding the time window to use for selecting case studies, a compromise usually has to be made between the quality of records/likely ability of researchers to recall their activities and the selection of grants whose outputs have had sufficiently long to develop [29]. The latter point was important in this study because the aim was to move beyond considering traditional outputs and also examine outcomes such as health gains. ARC instituted a new computerised database during the early 1990s and all their grants awarded since 1990 are held on this database. Prior to this, only paper records of unknown completeness were available. As an appropriate compromise between the various factors, we therefore decided to select grants that were awarded between 1990 and 1994.

### Selection of cases

Within a case study approach it is unlikely that the selection of cases will follow a straight-forward sampling logic in which those selected are assumed to be representative of a larger group [30]. Nevertheless, in adopting a multi-case approach the project aims to ensure not only that the benefits from the full range of modes of funding and types of research can be illustrated, but also that there is scope for some cross-case analysis. The selection of cases will, therefore, be somewhat purposive. Case studies based on four modes of funding will be included: institute grant, programme grant, project grant and fellowships. ARC-funded researchers will also be divided into three groups on the basis of their qualifications: basic researchers, clinical researchers, and Allied Health Professionals (AHP) such as physiotherapists. In their classic case study analysis of research utilisation, Yin and Moore [24] went to considerable lengths to ensure that they were including only studies where it was thought there had been utilisation. We do not propose to go that far, but, given that the idea is to illuminate the outcomes, it is considered desirable to concentrate on studies where it is thought there is a reasonable chance that there will be something to show. When examining the outcomes of research, even a stratified sampling approach is not thought to be sufficient because most impact usually comes from a small number of studies [23].

As a first step, we shall identify all publications in the relevant period from the principal investigators awarded ARC funds. Then, the researchers will be classified according to the journal impact factors (see below) of the journals in which their articles appear. The aim will be to draw up shortlists of possible researchers to include in the study: those in the top decile and those the middle of the range, with the final selection made on the basis of advice from ARC's Development Committee.

### Organisation of data collection

For case studies it is appropriate to use multiple sources of evidence converging on the same issues [4] and adopt a process of triangulation [27,30]. Three main interdependent methods will be used: documentary and literature review; semi-structured interviews with key informants; and bibliometric analysis. They will be applied in a partially overlapping way.

#### Documentary and literature review

We will read key project documents including the original research grant proposals, referees' reports and end of project reports. On the basis of the end of year reports from researchers, and the interviews (see below), we will also identify and read the core publications attributed to the research grant and any subsequent publications such as key citing papers, relevant clinical guidelines etc.

#### Semi-structured interviews with key informants

There will be about three interviews per case study. They will be based on a semi-structured interview schedule informed by the evaluation framework described below. They will, therefore, explore the origins of the research and the primary outputs such as the publications. In this way the initial list of publications identified as being related to the project will be refined. Furthermore, there will be a full exploration not only of the contribution to research training and career development, but also of any translation of the research findings into product development, policy and practice. In each case study the initial interviews will be with members of the relevant research team. Then snowballing techniques will be used to identify the people who might be able to provide most information about how the research has influenced subsequent research or been translated into product development, policy and practice.

#### Bibliometric analysis

Bibliometric approaches can play a useful role in the analysis of the research funded by specific biomedical research-funding bodies [19,31]. In the current analysis, the list of research papers published as a result of the project will first be refined as described above. Following that, bibliometric analysis will be conducted to record various matters including: the full funding acknowledgements; number of authors; citation counts; and comparison of number of citations with the journal impact factor of the publishing journals. This analysis will be conducted by a further part of the research team: those responsible for maintaining the ROD described above.

#### Clearance and validation

In every case a draft copy of the case study report will be sent to the principal investigator for comment. Such a step is an important part of the validation process and not just a matter of professional courtesy [24].

### Evaluation framework for ARC case studies

There are two elements in the evaluation framework adopted to organise the case studies being conducted in the assessment of the outcomes from ARC-funded research. Building on the framework developed by Buxton and Hanney [7,12], the two elements consist of a multi-dimensional categorisation of benefits from health research, and a model of how best to assess them. A logic model such as this helps facilitate assessment rather than pretending to be a precise model of how research utilisation occurs. The framework has been developed in various ways to meet the particular circumstances of ARC-funded research, which is often basic and investigator-led.

There are many steps involved in assessing outcomes from research. One of the key advantages in taking a detailed approach, such as that described below, is that it enables the issue of the counter-factual to be addressed. In other words, what would the world have looked like without the specific research being examined?

### The categories of payback

The multi-dimensional category of payback provides the evaluation criteria for the outputs and outcomes from ARC funding. The 5 main categories are:

a) Knowledge production

b) Research targeting, capacity building and absorption

c) Informing policy and product development

d) Health benefits

e) Broader economic benefits.

Each can be considered in turn, with various sub-categories explored and possible measures described.

#### Knowledge production

The knowledge produced by research is the first output and is contained in various publications and patent applications. Any type of publication can be considered, but it is generally thought that peer reviewed articles are the most important and, at least for biomedical research in industrialised countries, it is thought reasonable to assume that the overall output of research publications is fairly represented by peer-reviewed papers in international journals [19]. In addition to counting the number of publications, their quality and their impact can be assessed in various ways. The quality of knowledge production has traditionally been assessed by peer review, but various other methods can be applied. Papers that are accompanied by an editorial are often seen as being of particular significance. For those studies that are included in a systematic review there are now formal quality assessment techniques [32], as there are for reviews appearing in an overview [28].

Citation analysis can be applied to assess the impact the specific article is having within the research community [33,34]. Previous experience suggests that knowledge production will be particularly important for basic research, and certainly, on average, papers in basic research journals tend to be cited more frequently than ones in clinical journals [19,35].

A journal's 'impact factor' is based on the average number of times an article in the journal is cited; it can provide a short-hand version of citation analysis by giving some indication of the importance of the journal in which an article appears. The use of impact factors in analysis of biomedical research has been criticised [36] but, provided care is taken [37], it has been shown to be of some value [19].

Particularly when considering research that might be aimed at potential users outside the research community, it is often desirable to use a range of publication outlets including those journals with the highest readership among the groups at whom the research is targeted. In some fields these might well be journals that do not have an impact factor but are, nevertheless, significant as vehicles for dissemination of the knowledge produced [38-40].

#### Research targeting, capacity building and absorption

The better targeting of future research is frequently a key benefit from research, especially from research that is more basic and/or methodologically oriented. An indication of this comes from citation analysis. The enhanced targeting can be of the research conducted both by others and by the original researcher(s). Where follow-on research, especially by members of the original research team, is clearly associated with the original research it can be useful to obtain information on the source and amount of such funding [39]. As is developed in the paragraph below, one of the key roles of a medical charity can be to fund research in its field that will help to open up questions/issues that will then attract further funding from the general research funders such as the MRC and the Wellcome Trust.

Research training can be provided both as a result of the employment of staff on research projects and programmes, and through explicit funding for research training and career development [1]. One measure of research training, which may appear crude but has nevertheless been used in previous studies, is the number and level of higher or research degrees resulting, either totally or in part, from the research funding [1,14,39,41]. The career development of arthritis researchers goes much wider than specific training and is of considerable importance to ARC which aims to ensure that the pool of researchers in this field is a strong as possible. The reasoning is that this, in turn, should help ensure that arthritis as a topic is able to gain an appropriate share of the research funding available from general medical research funders. Some of ARC's funding schemes aim explicitly to provide career development, and for other researchers the receipt of a project grant from ARC can be important in advancing their career in research. Interviews can address this. Furthermore, they may also enable us to consider how far career development based on ARC funding helps propel some researchers into positions within the health-care system where they can play a role in ensuring that the later stages of translating research findings into outcomes are achieved.

#### Informing policy and product development

Research can be used to inform policymaking in a wide range of circumstances and the key issue is that policymaking involves those in positions of authority making choices that have a special status within the group to which they apply [27]. Policymaking is interpreted very broadly here and refers not just to national policies of the government, but also includes: policies made by managers at many levels within a health service; policies agreed at national or local level by groups of health-care practitioners in the form of clinical or local guidelines; policies developed by those responsible for training/education/inspection in various forms including training packages, curricula and audit and evaluative criteria [3]; and policies about media campaigns run by health-care providers. Basic research is less likely than that from clinical researchers or AHP to be used to inform policy. Various methods have been proposed for analysing the impact of research on health policymaking, including documentary review and interviews [26,27].

The position of systematic reviews is a little complex. They are themselves a form of research, but inclusion of a study in a systematic review is a form of secondary output and might lead on to further use.

At a similar level, although involving very different processes, research can also be used to inform product development [38]. Informing policies and product development are conceptually similar in that there generally has to be some subsequent adoption of the policy, or product, before the health and economic benefits can accrue [7].

#### Health benefits

Benefits in terms of health gains might be viewed as the 'real' payback or outcomes from health research. Greater effectiveness of health-care resulting from research-informed drugs or procedures should lead to increased health. Various measures of health gain exist, but for arthritis the emphasis, in most cases, is likely to be on those that assess reduction in pain or disability, and increase in mobility. While the benefits from arthritis research will not generally be measured in terms of life years gained, in some circumstances they might be captured by using Quality Adjusted Life Years (QALYs). This is often seen, in countries such as the UK, as a more appropriate approach than using Disability Adjusted Life Years (DALYs) [42]. There have been recent attempts to put a monetary valuation on the reduction in mortality and morbidity as a result of health research [16,43], but that is not being proposed for this study. At an overall level, it is possible that figures for the potential population who could benefit from the new drug or procedure could be identified, along with information about the level of benefit that individual patients might receive. If knowledge about adoption levels was then also taken into consideration it might be possible to indicate overall levels of benefit.

This category of benefits can be thought of as going wider than health gain, and some aspects can be seen as benefits to the health sector more generally. Cost savings in the provision of health-care may result from research-informed changes in the organisation of services or in the particular therapies delivered. It might be necessary to consider various issues here. These include whether potential savings have in practice been realised – either as cash savings or as the release of resources for other valuable uses [44]. Furthermore, it would be important to check whether costs are not simply being transferred elsewhere. Improvements could also arise in the process of health-care delivery and these could be measured by techniques such as patient satisfaction surveys [7].

#### Broader economic benefits

A range of benefits can accrue to the national economy from the commercial exploitation of research. These can take the form of employment and profits resulting from the manufacture and sale of drugs and devices [45]. The national economy could also benefit from exports and/or import substitution [46,47].

Whilst there is a danger of double counting, it is probably also important to adopt a human capital approach and focus on the value of production gained from having a healthy workforce. This can be measured by examining the reduction in days off work. Typically, in a human capital approach, potential future earnings are calculated for people who, as a result of advances in medical research, can continue to contribute to national production [14,15,48]. Those who use it, however, share the concerns that such an approach to assessing the benefits from research could have equity implications in that it would seem to favour research relevant for those of working age. This concern might be relevant here, in that many who suffer most from arthritis are retired, but reducing the days off work caused, for example, by low back pain, could be important. The economic burden of low back pain has been identified [49] and the potential role of research in reducing it was recently highlighted in a wide-ranging discussion of the benefits from medical research in the USA [50].

### Model for assessing the outputs and outcomes

**Figure 1:**
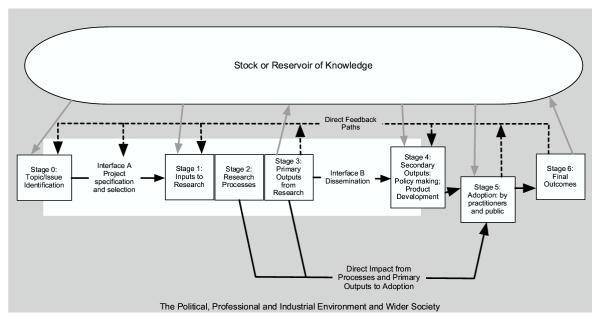
**Model for Organising the Assessment of the Outcomes of Health Research. **Sources: Adapted from previous versions of the Buxton/Hanney model for assessing the payback from health research [12,27].

The second element of the evaluation framework is the logic model. Its various stages are shown on Figure 1 and provide a way of organising the case studies. At least seven stages and two interfaces are identified and although they are presented in a linear form, the reality is much more complicated and there is also considerable feedback [7,12].

Stage 0: Topic/issue identification

Interface A: Project specification and selection

Stage 1: Inputs to research

Stage 2: Research processes

Stage 3: Primary outputs from research

Interface B: Dissemination

Stage 4: Secondary outputs – policymaking and product development

Stage 5: Adoption by practitioners and public

Stage 6: Final outcomes

While it is not possible totally to tie the categories of benefits to certain stages of the model, it is possible to identify broad correlations: categories a) and b) (knowledge and research benefits respectively) are together considered to be the primary outputs from research; category c) (informing policy and product development) relates to the secondary outputs; and categories d) and e) (health and broader economic benefits respectively) are the final outcomes. This approach can be incorporated into the analysis of each stage in turn as is set out below, where a few examples, drawn from the pilot studies, are used to illustrate how the framework seemed to be working in practice but could be refined in certain ways.

#### Stage 0: Topic/issue identification

The topic or issue identification stage covers the generation of the original ideas for the research. Its nature can vary considerably depending on whether the main driving force is internally generated by the researcher, or externally generated [27]. Most ARC funding falls into the former category: for many researchers the topics will be curiosity-driven and based on examination of the existing stock or pool of knowledge and opinions about where gaps, and/or opportunities, exist and further research could advance understanding. Such factors will also inform more clinical and AHP researchers, but here consideration of clinical needs could also be a factor and might be based on personal experience of treating patients, as became clear in the interview with the principal investigator in one of the case studies. Where research topics are externally generated, the identification of the issue comes from a process of needs assessment that could involve analysis either just within the scientific community or more widely. In the latter case, many groups could be involved. These include not only members of the wider research community and representatives of research funding bodies, but also potential users and beneficiaries of the research drawn from some combination of the wider political, professional, industrial and societal environment.

#### Interface A: Project specification and selection

The nature of the activities at Interface A will vary depending on the type of issue identification. Where the topics are externally generated, there are potential difficulties in ensuring both that the research community is actively engaged with the priorities that have been identified and that the project specification meets the needs as identified [27]. Where the issues are internally generated, the interface involves traditional processes of the researcher developing a detailed proposal and submitting it for peer review. Most of the issues are internal to the scientific world, but there is still a key interface between individual researchers and ARC as the research-funding body. Documentary analysis of ARC files provided information in the pilots that sometimes highlighted issues about how far the proposal was subject to changes as a result of the review process. It also proved useful, however, to supplement this with questions in the interviews.

#### Stage 1: Inputs to research

It can be important to consider not only the financial inputs, including any beyond the specific ARC funding, but also the experience of the research team and the knowledge base on which they built. Part of the idea behind examining any other funding brought in to support ARC research is again to see how far ARC funding is helping to facilitate the funding of arthritis research by general funders of health research: is ARC funding studies that produce findings that others believe are worth further investigation? The pilot studies confirmed that the complexities of identifying the exact funding streams behind any piece of research were best addressed by using a case study approach involving initial documentary review and following up issues in interviews. The pilots involved a case where other contributory funding contributed to what was clearly an ARC project, and therefore little attempt was made to portion out credit for outcomes to any funder other than ARC. In another case, however, the research was part of a stream of ARC-funded work and an effort was made to try to draw boundaries around what would be appropriate to include in the case study.

#### Stage 2: Research processes

Consideration can be given to how appropriate the proposed methods for a study turned out to be, and whether any difficulties were encountered. In some cases it could be relevant to explore how far potential users were involved at this stage. It is possible that difficulties identified at this stage could explain later problems with translation or uptake of the research findings.

#### Stage 3: Primary outputs from research

Knowledge production, as represented by the various types of publications, is a major primary output from the research. Various ways of measuring this were discussed above. The pilots also showed that the interviews used to refine the lists of publications from the specific funding in question, could also sometimes help to identify where non-conventional sources were being used as outlets for publications. Most of the primary outputs will feed into the stock of knowledge.

The research benefits in terms of targeting future research represent either feedbacks to further research conducted by team members, or findings that feed into the stock of knowledge and help target future research of others. An example from one pilot study showed not only how the principal investigator used her project to inform her own further work, but was also able to contribute to a much larger collaborative project. Interviews in another study showed that the research had informed considerable further work in industry, but as yet this had not led to any product development. Under the framework being used, it is possible to give that ARC-funded work considerable credit for informing the further research, but record its limited impact at the subsequent stages.

Capacity building can also be seen as a primary output. Accounts were given, in pilot study interviews, of the research training and higher degrees that resulted from the research.

#### Interface B: Dissemination

Dissemination is usually seen as being somewhat more active than the mere production of academic publications containing the knowledge. There are, however, clear overlaps between some activities. Sometimes it is possible to record not just dissemination activities but also the successful transfer of research findings to potential users in the political, industrial, professional environment and wider society. Previous analyses of how to increase the implementation of research findings [28] will help inform the issues being examined in the case studies at the dissemination and later stages. Presentations to potential academic and user groups, and media activities, are major ways of disseminating findings, as are the production of brief summaries of findings targeted at specific user groups. In previous case studies, attention has also focused on the way some researchers conduct study days, or training, based on the approach developed by their research and these can be highly effective dissemination mechanisms [51]. The pilots provided an example of the importance of this and, indeed, of the role of individual researchers in networking and disseminating information.

#### Stage 4: Secondary outputs–policymaking and product development

As noted above, policymaking and product development activities can result in a wide range of secondary outputs, and various methods are needed to identify research-informed policies. In one case study, a review of a database revealed that one project had been cited in a clinical guideline unbeknown to the research team, whereas in another pilot it took interviews to identify that the research was informing local guidelines and care pathways. The use of the research in systematic reviews was also revealed in various ways in the pilot studies. Where the research seems to have resulted in secondary outputs it is useful to explore the factors that have led to this.

In relation to product development, if research findings are incorporated into the process of developing a product, for example a new drug for arthritis, this can be seen as an important secondary output. In the preliminary set of interviews, most people referred to how ARC-funded research had played a key role in the production of anti-TNF therapy for arthritis. In a pilot study, interviews revealed the extent to which industry's attempts to use one stream of research for product development had not, so far, been successful.

#### Stage 5: Adoption by practitioners and public

For the research findings incorporated into secondary outputs to result in final outcomes there usually has to be some behavioural change by practitioners, and/or the public. This may involve take-up of new drugs or procedures as set out in a secondary output such as a guideline from the National Institute for Clinical Excellence (NICE). Sometimes the adoption comes as a direct result of the primary outputs, as when clinicians – often at the cutting edge – decide to implement research findings even prior to the development of clinical guidelines. Either way, it is important to try to establish the adoption or take-up rates and to explore how far the behavioural change can be attributed to the specific research findings, as opposed to other factors such as a more general change in climate of opinion in relation to, for example, the importance of exercise. In one pilot study where interventions based on research filtered into practice, a series of interviews was used to attempt to identify both the precise role of the specific ARC-funded project and possible levels of uptake.

The role of the public in responding to informed advice – often research-based – is seen as increasingly important, especially in a field such as arthritis [52]. Various factors can be explored here. These include the extent to which patient behaviour might change as a result of interactions with health-care providers who promote research-based messages, and how far the public might respond directly to publicity about research findings when they are used, for example, in media campaigns encouraging participation in preventative activities [28].

#### Stage 6: Final outcomes

The final outcomes are the health and broader economic benefits identified in categories d) and e) above. These are increasingly seen as being the ultimate goal of health research funding, but their precise estimate in practice often remains difficult [5-7]. In one pilot study, it was possible to produce audit figures from one area where there is known to have been local implementation of the research findings.

### Planned analysis and synthesis

Each of the 16 cases will be written up as a narrative organised according to a common structure based on the various stages of the logic model. Each study should potentially, therefore, provide illumination as to the processes that could lead to outcomes and illustrations of such outcomes. In addition, the common structure of each case should facilitate some cross-case analysis that will not only look for common factors associated with research that has led to outcomes, but also see how far such outcomes are associated with different modes of funding and types of research. Some of this analysis should be based on the previous findings that are embedded into the evaluation framework: for example, basic research might be expected to produce a reasonable number of knowledge outputs but be less likely than clinical or AHP research to inform policies. Some other aspects of the analysis, however, are likely to be exploratory: detailed analysis of factors related to the role of medical charity research in contributing to outcomes appear, as yet, not to be well established.

## Conclusions

This paper sets out the aims and methods to be adopted in an innovative study to review the outcomes of the research funded by the Arthritis Research Campaign, one of the leading medical charities in the UK. At a time of growing emphasis on both accountability and evidence-based policy making, it is important for research-funding bodies to be able to show the results of their funding and base their policies on analyses of the processes involved in producing outcomes [53]. Based on the results of the piloting, a decision was made to go ahead with the full study.

Finally, one of the challenges for the future will be to operationalise such analysis on a regular, and therefore less resource intensive, manner. It is hoped that the study will also shed light on these practical considerations, and do so in a way that will enable a system to be developed that meets the specific needs of the particular research funding body [4], in this case ARC.

## Competing interests

The work was funded by ARC.

## Authors' contributions

JG led the design of the study, with all authors making contributions. SH drafted the article with contributions from all authors.
